# Editorial: Developments in sickle cell disease therapy and potentials for gene therapy

**DOI:** 10.3389/fmed.2023.1208192

**Published:** 2023-06-14

**Authors:** Robert W. Maitta, Hollie M. Reeves, Magali J. Fontaine

**Affiliations:** ^1^Department of Pathology, Case Western Reserve University, Cleveland, OH, United States; ^2^Department of Pathology, University of Maryland, Baltimore, MD, United States

**Keywords:** sickle cell disease, therapy, transplantation, complications, review

Since first described, sickle cell disease (SCD) has represented a therapeutic challenge for clinicians trying to help patients enduring the ramifications of this dreadful condition. This life-long disease from the first crisis begins with processes that will change the physiological milieu of patients. These processes in the setting of chronic anemia can translate into potentially worsening neurological symptomatology and organ damage that characterize those patients having frequent and recurrent vaso-occlusive crises. Thus, it is readily apparent that reducing the negative effects caused by levels of hemoglobin S does represent the best approach to ameliorate the long-term outlook of patients. The main therapeutic is to provide chronic transfusion support of patients with SCD. However, many patients are unable to tolerate the known adverse events of concomitant chelation therapy needed to reduce the iron load caused by a long transfusion issue, we introduce a variety of SCD-related topics describing concepts and advances in the treatment of this disease.

Most SCD patients manifest symptoms and complications of the disease from childhood. With this in mind, this issue includes a large study from French Guiana describing the incidence and type of complications of pediatric patients over time, and determining the incidence of complications such as acute chest syndrome and ischemic stroke (Gargot et al.). Importantly, even though these data show that the risk of ischemic stroke was low at 3.1%, this risk than doubled by the time patients reached teenage years. These data emphasizes that therapeutic approaches for SCD patients should focus on targeted interventions early in childhood to decrease complications. For diagnosis and testing, this issue also revisits both erythrocyte and reticulocyte counts of SCD patients as predictors of vasoocclusive crises (Feugray et al.). Authors of this study recommend the use of reticulocyte parameters obtained with a complete blood count. Specifically, a higher reticulocyte count in conjunction with higher medium reticulocyte fluorescence have the highest sensitivity and specificity (81% and 88% respectively) for predicting a looming crisis.

Additionally, the current issue includes a multicenter study from Italy looking at over 1,500 SCD patients treated with hydroxycarbamide and different transfusion regimens (Graziadei et al.). This study indicates that a proportion of SCD patients continued to experience symptoms requiring additional intense chronic transfusion support with no reduction in acute SCD-related complications despite receiving hydroxycarbamide. Furthermore, this study exemplifies one of the known complications of transfusion therapy in SCD and that is alloimmunization, which occurred in 8.5% of patients despite matching for Rh and Kell antigens. The authors argue that differences between red blood cell donors and patients explain the high alloimmunization rates seen in SCD patients (1).

Among the known complications of SCD, avascular necrosis of the hip and shoulder are often requiring surgical interventions in this patient population, as symptomatology of the affected joint(s) worsen and crises accumulate. In this Research Topic the use of intense hyperbaric oxygen therapy will be introduced and discussed for SCD patients with avascular necrosis of hip and/or shoulder, that can lead to resolution of the necrosis (Alshurafa, Elhissi et al.). The risk of iron overload, a major complication of chronic transfusions in SCD is being mitigated by performing exchanges instead of regular red cell transfusion. But despite good patient compliance, chronic red cell exchanges did not extend the life expectancy of patients with marked iron overload (Zhou et al.). Moreover, an article in this Research Topic will review the role that systemic hypertension plays in SCD patients in Cameroon by outlining a deep analysis of blood pressure variables that affect renal function over time (Nguweneza et al.).

Lastly, the current issue includes a comprehensive review of most SCD disease-modifying therapies including hydroxyurea, L-glutamine, voxelotor, and crizanlizumab known to reduce pain crises (Tanhehco et al.). Matched-related and haploidentical hematopoitic stem cell transplant (HSCT) with modified conditioning regimens will be compared to and contrasted to new gene therapies entering clinical trials in the same review. Understanding that adverse events to biologicals can also occur, an article proposes how to triage patients presenting with reactions to crizanlizumab while still being beneficial to the patient's treatment outcome [Alshurafa and Yassin (a)]. One of the studies in this Research Topic reviews L-glutamine's role in reducing oxidative stress in SCD over an extended follow-up period (Elenga et al.) and confirms the medication's safety while it reduces hospitalizations, need for transfusions and organ damage. Similarly, this issue also describes the high effectiveness of voxelotor in SCD patients with significant kidney disease [Alshurafa and Yassin (b)].

As mentioned above, allogeneic HSCT is a potentially curative, therapy is limited since finding suitable HLA compatible donors is the first challenge that needs to be overcome. This type of transplantation and graft manipulation including T cell depletion, presents with high infection risks secondary to transplantation conditioning regimens, as well as risks of graft vs. host disease (Bhalla et al.). Despite the potential for allogeneic HSC transplantation, use of new gene-editing tools such as Clustered Regularly Interspaced Short Palindromic Repeats (CRISPR) and gene transfer technologies to correct the genetic cause of the disease are gaining momentum. CRISPR-associated protein currently represents an opportunity to treat or even cure patients with SCD by targeting either B-cell lymphoma/leukemia 11A gene (BCL11A) and the promoter regions of gamma globin genes (HBG1/2), both of which have been identified to significantly increase HbF protein expression. This issue presents a meta-analysis of the most recent work showing that of these two genes, HBG1/2 has the greater effect on HbF induction (Quagliano et al.). This Research Topic also contains a novel study using the Townes SCD mouse model that shows the extent by which the microRNA29B can induce HbF production *in vivo* (Gu et al.). This report demonstrates that this induction occurs by silencing the *MYB* gene product. Along these lines another article in this issue outlines challenges facing scientists trying to find new HbF-inducing agents and will discuss recently completed or ongoing clinical trials testing some of these agents (Pavan et al.). Additionally, a review in this issue will describe the effects of homology-directed repair of the *HBB* gene, and disruption of *cis*regulatory elements of BCL11A or leukemia/lymphoma related factor binding sites in the γ-globin gene promoters that result in enhancement of HbF expression (Zarghamian et al.). Finally, an article in this Research Topic will describe how inhibition of BACH1 transcription factor using a novel small molecular inhibitor can increase the concentration of HbF and even enhance the effectiveness of hydroxyurea in the setting of drug resistance using *in vitro* and *in vivo* models (Belcher et al.).

In conclusion, approaches to treat and cure SCD are rapidly developing. This is a period of great excitement and hope. Contributors to this Research Topic and the editors of this special topic encourage readers to appreciate this collection as an attempt to provide up to date information while introducing a variety of research areas in the field. Clinical advances including current clinical trials will be covered with focus on all recent developments in the field.

## Author contributions

All authors listed have made a substantial, direct, and intellectual contribution to the work and approved it for publication.
